# Establishment of the Certified Research Ethics Professionals: An Ethical Review Expert
―Translated in English from Japanese Version―

**DOI:** 10.31662/jmaj.2021-0056

**Published:** 2021-09-27

**Authors:** Yusuke Ebana, Sou Hee Yang, Megumu Yokono, Masayuki Yoshida

**Affiliations:** 1 Life Science and Bioethics Research Center, Tokyo Medical and Dental University (TMDU), Tokyo, Japan; 2 Graduate School of Social Sciences, Waseda University, Tokyo, Japan; 3 School of Social Sciences, Waseda University, Tokyo, Japan

**Keywords:** Research ethics, Research ethics committee, Certified Research Ethics Committee Professionals, CReP, Medical guideline, Clinical trials act, Certified review board

## Abstract

Medical research is indispensable to develop new treatments or diagnostic methods. An ethics review board reviews the validity of such medical research. However, with the recent advances in medicine, a meaningful review of medical research often requires advanced knowledge. There is thus a growing necessity for a professional who can support ethical review. Therefore, a new system called the Certified Research Ethics Committee Professional (CReP), an Ethical Review Expert, has been established.

## Introduction

There have been remarkable advancements in medical technology in recent years. Knowledge in the fields of medical genetics and molecular biology is being updated day after day, with flowing suggestions for new treatment methods. It is now possible to provide targeted therapy for cancer or rare and intractable diseases by screening genetic information, the characteristics of cancerous cells and compounds. Many patients also benefit from the advancement of optics and catheter technology, such as an endoscopic operation, which has enabled them to receive minimally invasive treatment instead of open-heart surgery or laparotomy. However, it is undeniable that various unethical studies have been found in the history of clinical research ^(1), (2)^.

Numerous medical research and clinical trials have born such benefits to patients. After conducting preclinical research involving basic experiments on cells or animals, those with confirmed efficacy and safety may be used on humans as a new treatment method. A researcher who wants to propose a new kind of treatment should follow certain procedures, one of which is going through an ethics and clinical trial reviews. The requirement for the reviews are based on the “Ethical Guideline for Medical Research Involving Human Subjects” (Ministry of Education, Culture, Sports, Science and Technology and Ministry of Health, Labor and Welfare)^(3)^, “Ethical Guidelines for Human Genome and Genetic Analysis Research” (Ministry of Education, Culture, Sports, Science and Technology; Ministry of Health, Labor and Welfare; and Ministry of Economy, Trade, and Industry)^(4)^, and “Clinical Trials Act” (Ministry of Health, Labor and Welfare)^(5)^.

In such a complicated situation, we established a system called the Certified Research Ethics Committee Professional (CReP), a group of experts in ethical review. In this article, we described the role of CReP in ethical review. This article is an English version of a paper already published in Japanese ^(6)^. The Editors-in-Chief of JMA Journal have permitted the submission of this manuscript.

## Ethics Review

As stated in the Declaration of Helsinki, when providing new medical treatment to patients, a third party must review the scientific reasonableness and theoretical validity of the treatment, even when there is an agreement between the medical professional who provides the treatment and the patient who receives the treatment that provides for the patient’s consent^(7)^. This requirement is established to ensure that the research subjects do not suffer from disadvantages stemming from information asymmetry. The abovementioned guidelines provide that the “research ethics committee” or the “institutional review board” (IRB) is responsible for the review. When conducting a research or clinical trials, the principal investigator should prepare a research protocol and other required documents, including the instructions for the research subjects and the procedure manual necessary to conduct the research, in accordance with relevant laws and guidelines.

On the other hand, to protect the research subjects or promote scientific reasonableness of the research, the members of the ethics review committee examine the validity of conducting a research based on the submitted documents. The committee reviews the scientific reasonableness and the ethical validity while also ensuring that the research is in compliance with relevant laws and guidelines. However, it is not necessarily true that not only the researchers but also the committee members are aware of the details of the standard procedures, such as the review application. Even if the researchers have sufficient knowledge about the treatment and research in their fields of expertise, they often require support to comply with relevant laws and guidelines related to such research or treatment. Moreover, the research ethics committee board members in many institutions are also engaged in their own tasks or research as experts in their fields, and while they have profound knowledge and experience in their own fields of research, conducting ethics review is merely one of the various duties of many of those working in universities, hospitals, and companies.

Research institutions should make efforts to ensure the reliability and safety of the research so that it does not create disadvantages for the subjects. However, as the laws and guidelines do not always provide detailed instructions, handling each case, in reality, is not an easy task for the institutions. Moreover, as mandated by the legal regulations on clinical research, a Certified Review Board (CRB) needs four dedicated full-time supporting staff, increasing the importance of the support department. For these reasons, there is an increasing demand for fostering experts who are knowledgeable about clinical research and ethics review.

In Japan, office clerks often take on the roles of the research ethics committee board members in many institutions and often review ethics while also working on other assignments. Additionally, the committee’s uniform management and operation become challenging, as employee transfers occur once every few years.

## CReP: Ethical Review Expert System

Since 2016, as a part of its research integrity project, the Japan Agency for Medical Research and Development (AMED) has supported fostering and training of ethics review experts called CRePs. CRePs are professionals who promote the protection of research subjects and fair research practice. In the United States, there have long been certified IRB professionals called CIPs^(8),(9), (10)^. CIPs are a group of experts who runs the IRB, that is, the ethics review committee within a research institution, and they aim to improve the quality of the Human Research Protection Program. More specifically, CIPs support the ethics review committee or IRB members with their knowledge on laws, regulations, principles, and guidelines in the United States, as well as general knowledge about ethics. Most IRBs have a CIP, and there are more than 3,000 CIPs in the United States. The collaboration between CReP in Japan and CIP in the United States is a critical initiative. In recent years, more international joint research has been conducted than ever before. Of course, multilateral clinical trials under GCP for regulatory approval are also necessary. The existence of CReP and CIP, who are aware of the circumstances of each country, will become more crucial for the smooth execution of international joint research.

In many Japanese institutions, nonmedical clerks check the validity of the content and the insufficient description in the research protocol and the related documents before the review by the REC board member according to the rules of each institution. Since there is no detailed description of ethical review and research implementation in laws and guidelines, each institution often operates based on their own interpretations. Filling the gaps between the texts of laws and guidelines and making efforts to have a common understanding are necessary. Those who are actually involved in ethical review need to have a common view. Therefore, we created a system to certify those who have the necessary knowledge in ethical review as professionals. By summarizing the common items necessary for professionals as core competencies, we aimed to standardize the clerical work of ethical review. Table 1 shows a list of core competencies required for CRePs regarding their knowledge about medical guidelines, genome guidelines, and legal regulations on clinical research, to provide proper support for researchers and the ethics review committee. These competencies would enable CRePs to successfully promote the protection of the research subjects and fair research practice. The education materials are also being prepared so that CRePs can continue their education after acquiring the CReP qualification. As the first example of CReP’s efforts, a working group was formed among the members to create a unified format for the new ethical guidelines. Second, efforts have begun to illustrate best practices in ethical review procedure. We believe that both are milestones for the standardization of ethical review.

**Table 1.** Core Competencies for CReP.

**Major item 1**: Understanding the outline of related laws and guidelines
**Middle item A**: Ethical Guidelines for Medical and Health Research Involving Human Subjects
**Middle item B**: Ethical Guidelines for Human Genome/Gene Analysis Research
**Middle item C**: Scope of application of the Clinical Trials Act
**Middle item D**: Scope of application of the Order for Enforcement of the Act on Securing Quality, Efficacy and Safety of Products Including Pharmaceuticals and Medical Devices
**Middle item E**: Scope of application of the Act on Securing Safety of Regenerative Medicine
**Middle item F**: Norms such as the Declaration of Helsinki and Belmont Report
**Middle item G**: About past cases
**Middle item H**: Update of the latest information on each related laws/guidelines
**Major item 2**: Committee management support
**Middle item A**: Membership requirements (sciences, law/humanities, general position), committee establishment requirements
**Middle item B**: Support for researchers (until the start, amendment, stop, and end of research)
**Middle item C**: Committee holding procedure
**Middle item D**: Cooperation with the Conflict of Interest Committee/Secretariat
**Middle item E**: Cooperation with ethics examination-related departments
**Middle item F**: Correspondence to external surveys, etc.
**Major item 3**: Committee document preparation/storage and contract
**Middle item A**: Preparation of materials used by the committee
**Middle item B**: Preparation of committee meeting minutes
**Middle item C**: Examination result notification
**Middle item D**: Confidentiality pledge of committee members/Conflict of Interest
**Medium item E**: Procedures required for contracts (clinical trials and clinical research)
**Middle item F**: Storage of committee-related documents
**Middle item G**: Disclosure of committee member list and minutes
**Major item 4**: Correspondence to individual cases
**Middle item A**: Judgment that the guideline is not applicable
**Middle item B**: Judgment of quick review
**Middle item C**: Correspondence to deviation
**Middle item D**: Response to adverse events and compensation insurance
**Middle item E**: Responding to complaints from research subjects
**Major item 5**: Understanding the status of research implementation
**Middle item A**: Implementation status report (status of obtaining consent to participate in research, status of continuation, etc.)
**Middle item B**: Monitoring/audit
**Middle item C**: Safety report
**Middle item D**: Report of adverse events
**Major item 6**: Implementation and recording of education and training for the committee secretariat, researchers, institution heads, and committee members
**Middle item A**: Education and training for the committee secretariat, researchers, institution heads, and committee members
**Middle item B**: Records related to education/training implementation, records of training for committee members

## Future Outlook

Improving the treatment quality for clinical research, especially for certain types of clinical research, is crucial. Expert knowledge is indispensable for increasing the safety and trustworthiness of such treatments. Until now, there has been a limited number of communities for such professionals. By providing information-sharing opportunities for those with CReP qualifications, the quality of support for clinical research will improve and become standardized.

By certifying a nonmedical clerk as a professional, a community to which the new professional belongs to was created, and a new network was built. Through this network, professionals can collaborate to exchange and share information on the outline and procedures of actual ethical reviews that they know. Consequently, the knowledge and procedures of the research ethics committee will be standardized, thus also standardizing the research ethics committee’s review. However, it is hard to say that the standardization of ethical examination has been realized yet. CRePs are concentrated in the core institutions of each region, such as core clinical research hospitals, university hospitals, and national centers, and act as leaders in the region. According to the 2019 Static/Dynamic Survey of Medical Institutions and Hospital Report of the Ministry of Health, Labour and Welfare of Japan, there are 401 hospitals with more than 500 beds in Japan (data not shown, only Japanese). Since it can be inferred that many clinical studies are conducted in large hospitals to which doctors and researchers who present their research at each academic meeting belong to, it is desirable that CRePs be also assigned to each institution.

## Conclusion

This paper has reviewed the ethical review necessary for conducting clinical research and the new system for CRePs, the ethics review experts. The CReP Accreditation Committee currently accredits 180 people who supports the ethical review of each institution. For the proper implementation of medical research practice, each facility should protect the research subjects and improve research integrity by creating positions for such qualified professionals. For certain types of clinical research that carry high risks, support from qualified experts would become all the more essential.

## Article Information

### Conflicts of Interest

None

### Sources of Funding

This work was supported by Research and Development of Model Material/Program for Research Integrity Education from Department of Research Integrity and Legal Affairs, Japan Agency for Medical Research and Development (AMED). AMED No. 1E407 and 1E410.

### Acknowledgement

We would also like to express our gratitude to the project participants, supporters, and research staff at TMDU and associated hospitals. We also thank the Department of Research Integrity and Legal Affairs, Japan Agency for Medical Research and Development (AMED), for their efforts and financial support. This manuscript has already been published in Japanese language (Journal of New Remedies & Clinics; ISBN0559-8672). The journal has given permission to publish the English version. We would like to thank the journal editor-in-chief. The original version is available at https://www.iyaku.info/magazine/?p=1&sf=1&ca3=67&ca4=10.

### Author Contributions

YE planned and conducted all studies. MY was involved in research planning advice. SY and MY were involved in assisting in writing this article.

### Approval by Institutional Review Board (IRB)

It is not applicable because it does not correspond to “Medical and Health Research Involving Human Subjects.”
